# Analysis of the Complete Genome Sequence of the Widely Studied Strain Bradyrhizobium betae PL7HG1^T^ Reveals the Presence of Photosynthesis Genes and a Putative Plasmid

**DOI:** 10.1128/MRA.01282-19

**Published:** 2019-11-14

**Authors:** Sylvie Cloutier, Eden S. P. Bromfield

**Affiliations:** a Ottawa Research and Development Centre, Agriculture and Agri-Food Canada, Ottawa, Ontario, Canada; Indiana University, Bloomington

## Abstract

Here, we present the complete genome sequence of the widely studied strain Bradyrhizobium betae PL7HG1^T^, isolated from a tumor on the roots of sugar beet. The genome consists of a 7.2-Mbp circular chromosome containing key photosynthesis genes but not genes for nodulation and nitrogen fixation. A putative plasmid was also detected.

## ANNOUNCEMENT

The genus Bradyrhizobium consists of diverse species of bacteria and includes members that possess genes for photosynthesis and/or symbiotic association with legume plants. Based on genomic analyses, we recently described the species Bradyrhizobium symbiodeficiens (1), which consists of strains that lack key genes for nodulation and nitrogen fixation.

The widely studied strain Bradyrhizobium betae PL7HG1^T^, isolated in 2004 from a tumor on the roots of sugar beet by Rivas et al. (2), currently represents the only other named species in the genus *Bradyrhizobium* in which nodulation and nitrogen fixation genes have not been detected. To make phylogenomic comparisons with our strains, we sequenced the complete genome of the reference strain PL7HG1^T^, obtained from the BCCM/LMG culture collection (number LMG 21987). Bacteria from pure culture were grown aerobically on yeast extract mannitol agar medium at 28°C (3). Genomic DNA was extracted using the Promega Wizard SV genomic DNA purification system and cleaned with a Qiagen DNeasy PowerClean Pro kit. Sequencing was done at the Genome Quebec Innovation Centre, Canada, using the Pacific Biosciences (PacBio) RS II single-molecule real-time (SMRT) platform (4, 5). Using two SMRT cells, a total of 141,096 polymerase reads with an average read length of 13,372 bp were generated; the estimated genome coverage was 175-fold.

Reads were *de novo* assembled with the Hierarchical Genome Assembly Process (HGAP) (6). Quality control was performed by aligning short subreads on long subreads with Basic Local Alignment with Successive Refinement (BLASR) (7). Assembly was performed with the Celera Assembler (8) and the result polished with Quiver (6). Default settings were used for all software.

The complete genome of B. betae PL7HG1^T^ comprises a circular chromosome of 7,150,095 bp and a putative plasmid (pBbPL7HG1) of 269,307 bp, with average GC contents of 64.8% and 60.6%, respectively.

The chromosome was found to contain a total of 7,167 genes, 7,113 coding sequences, 47 tRNAs, and a single rRNA operon, using the NCBI Prokaryotic Genome Annotation Pipeline (PGAP-4) (9, 10). Analyses performed with the Pathosystems Resource Integration Center (PATRIC) v. 3.5.26 platform (11) indicated that the most abundant genes were those involved in metabolism (936 genes), energy (316 genes), membrane transport (243 genes), and protein processing (233 genes). The chromosome of PL7HG1^T^ does not contain a symbiosis island or key nodulation (*nodDYABCSUIJ*) and nitrogen fixation (*nifDKEN*, *nifH*, *nifA*, and *fixABCX*) genes. Genes for type I and II/IV secretion systems were found but not those for type III secretion systems.

A photosynthetic gene cluster (PGC) of about 51 kb was detected that contained photosynthesis genes encoding the light-harvesting protein beta and alpha subunits (*pufBA*), reaction center L, M, and H subunits (*pufLM* and *puhA*), bacteriochlorophyll (*bchIDOCXYZGPFNBHLM* and *acsF*), carotenoid (*crtIBCDEF*), photosynthesis repressor proteins (*ppsR1* and *ppsR2*), and bacteriophytochrome (*bphP*). The genetic organization of the PGC is nearly identical to that in Rhodopseudomonas palustris CGA009 (12), Bradyrhizobium sp. strain S23321 (13), and Bradyrhizobium amphicarpaeae 39S1MB^T^ (14).

Based on BLAST searches using the PLSDB database (15), the putative plasmid pBbPL7HG1 has 99.5% similarity (79% coverage) to a 285,478-bp plasmid (GenBank accession number CP025114) in *Bradyrhizobium* sp. strain SK17. Detected in pBbPL7HG1 were plasmid replication initiation protein genes (*repABC*). Genes for antibiotic resistance, heavy metal resistance (cobalt, zinc, and cadmium), and carbon monoxide oxidation (*coxGFEDLSMC*) were also found.

Further analysis of the PL7HG1^T^ genome will be useful for studies on the evolution and taxonomy of the genus *Bradyrhizobium*.

### Data availability.

This whole-genome shotgun project has been deposited at DDBJ/ENA/GenBank under the accession numbers CP044543 (chromosome) and CP044544 (putative plasmid pBbPL7HG1). Raw PacBio data have been deposited in the NCBI Sequence Read Archive under the BioProject accession number PRJNA575551.
